# Involvement of miR-518c-5p to Growth and Metastasis in Oral Cancer

**DOI:** 10.1371/journal.pone.0115936

**Published:** 2014-12-23

**Authors:** Makoto Kinouchi, Daisuke Uchida, Nobuyuki Kuribayashi, Tetsuya Tamatani, Hirokazu Nagai, Youji Miyamoto

**Affiliations:** Department of Oral Surgery, Subdivision of Molecular Oral Medicine, Division of Integrated Sciences of Translational Research, Institute of Health Biosciences, Graduate School of Tokushima University, Tokushima, Japan; Johns Hopkins University, United States of America

## Abstract

We have previously demonstrated that a stromal cell-derived factor-1 (SDF-1; CXCL12)/CXCR4 system is involved in the establishment of metastasis in oral cancer. Recently, small non coding RNAs, microRNAs (miRNAs) have been shown to be involved in the metastatic process of several types of cancers. However, the miRNAs that contribute to metastases induced by the SDF-1/CXCR4 system in oral cancer are largely unknown. In this study, we examined the metastasis-related miRNAs induced by the SDF-1/CXCR4 system using B88-SDF-1 oral cancer cells, which exhibit functional CXCR4 and distant metastatic potential *in vivo*. Through miRNA microarray analysis, we identified the upregulation of miR-518c-5p in B88-SDF-1 cells, and confirmed the induction by real-time PCR analysis. Although an LNA-based miR-518c-5p inhibitor did not affect cell growth of B88-SDF-1 cells, it did significantly inhibit the migration of the cells. Next, we transfected a miR-518c expression vector into parental B88 cells and CAL27 oral cancer cells and isolated stable transfectants, B88-518c and CAL27-518c cells, respectively. The anchorage-dependent and -independent growth of miR-518c transfectants was significantly enhanced compared with the growth of mock cells. Moreover, we detected the enhanced migration of these cells. The LNA-based miR-518c-5p inhibitor significantly impaired the enhanced cell growth and migration of miR-518c transfectants, indicating that these phenomena were mainly dependent on the expression of miR-518c-5p. Next, we examined the function of miR-518c-5p *in vivo*. miR-518c transfectants or mock transfectants were inoculated into the masseter muscle or the blood vessels of nude mice. Tumor volume, lymph nodes metastasis, and lung metastasis were significantly increased in the mice inoculated with the miR-518c transfectants. These results indicated that miR-518c-5p regulates the growth and metastasis of oral cancer as a downstream target of the SDF-1/CXCR4 system.

## Introduction

We previously demonstrated that B88 cells, which are oral cancer cells that express the chemokine receptor CXCR4, specifically metastasize to cervical lymph nodes via a stromal cell-derived factor (SDF)-1 gradient produced by the lymphatic stroma [1]–[3]. The forced expression of SDF-1 in B88 cells (named B88-SDF-1 cells) conferred enhanced cell motility and lung metastasis following intravenous inoculation [4]. Recently, we also demonstrated that CXCR4 expression contributes to the metastatic potential of salivary gland cancers [5]. Furthermore, we have also demonstrated that blocking CXCR4 with 1,1′ -[1,4-Phenylenebis(methylene)]bis-1,4,8,11-tetraazacyclotetradecane octahydrochloride (AMD3100), a CXCR4 antagonist, may be a potent anti-metastatic therapy for CXCR4-related head and neck cancer [5], [6].

Although the SDF-1/CXCR4 system mainly functions as a chemotactic factor in cancer cells to seed metastatic sites, recent studies demonstrated that this system also regulates tumor growth, cancer cell-tumor microenvironment interaction and angiogenesis [7]–[10]. In fact, to establish the metastases, several important processes, such as invasion, intravasation, extravasation and ectopic growth potential, are indispensable. Thus, it is critical to investigate the function of downstream target(s) responsible for the process of metastasis in the SDF-1/CXCR4 system. microRNAs (miRNAs) are small regulatory non-coding RNAs that bind to specific target mRNAs, leading to translational repression [11]. miRNAs are involved in the regulation of biological processes, including cell growth, differentiation and apoptosis, both in physiological conditions and during diseases, such as cancer [11]. Recent evidence has indicated that miRNAs play crucial roles in the metastatic process of several types of cancers [11]. However, the miRNAs that contribute to metastases induced by the SDF-1/CXCR4 system in oral cancer are largely unknown. Thus, in this study, we examined target miRNAs regulated by the SDF-1/CXCR4 system in B88-SDF-1 cells using miRNA microarrays and investigated their functional role on metastasis in oral cancer.

## Materials and Methods

### Ethics statement

The mice were handled in accordance with the recommendations in the Guide for the Care and Use of Laboratory Animals of the National Institutes of Health. The protocol was approved by the Animal Research Committee, Tokushima University (Permit Number: 11111). Briefly, all mice were housed under pathogen-free condition, received food and water ad libitum, and maintained in a 12 h light/dark cycle in an appropriate temperature-controlled room. All surgery and euthanasia were performed under sodium pentobarbital anesthesia, and all efforts were made to minimize suffering. B88 cells [1], [12] were originally established from a patient with tongue cancer in 1988. The Ethics Committee of the Tokushima University Hospital waived the need for consent on the use of this cell line (Permit Number: 453).

### miRNA microarray analysis

Small RNA for miRNA microarray analysis was extracted from serum-starved B88-mock cells and B88-SDF-1 cells. A miRNA microarray was performed and analyzed in TORAY (Tokyo, Japan) using a human 3D-Gene Array (V16.1.0.0). The microarray raw data were deposited in Gene Expression Omnibus (GEO, http://www.ncbi.nlm.nih.-gov/geo) according to minimum information about microarray experiment (MIAME) guidelines. The accession number is GSE59323.

### Cells and cell culture

Oral cancer cells were deemed free of mycoplasma and bacterial contaminants. CAL27 cells are oral cancer cells that were obtained from the American Type Culture Collection (Rockville, MD). B88 cells highly metastasize to cervical lymph nodes, when the cells are inoculated in the masseter muscle of nude mice, but rarely metastasize to lungs by the intravenous inoculation [1], [4]. In contrast, CAL27 cells rarely metastasize to cervical lymph nodes or lungs, when the cells are inoculated into the masseter muscle or tail vein of nude mice, respectively (unpublished observation). The cells were maintained in Dulbecco's Modified Eagle Medium (DMEM; Sigma, St. Louis, MO) supplemented with 10% fetal calf serum (FCS), 100 µg/mL streptomycin, and 100 U/mL penicillin in a humidified atmosphere of 95% air and 5% CO2 at 37°C.

### Mice and *in vivo* study

BALB/c nude mice were purchased from CLEA Japan (Osaka, Japan). The mice were maintained under pathogen-free conditions. The experiments were initiated when the mice were 8 weeks of age and were performed as described previously [1], [4]. Briefly, cells were orthotopically inoculated into the masseter muscle of nude mice (2×10^6^) or inoculated into the blood vessels of nude mice (1×10^6^); those mice were sacrificed at day 35. The tumor volume was estimated by measuring the tumor size and using the following formula: tumor volume  = 1/2 × L × W^2^, where L and W represent the largest diameter and the smallest diameter, respectively. The presence or absence of lymph node and distant metastases was confirmed by hematoxylin and eosin staining.

### Transfection

Cells (5×10^5^ cells/dish) were seeded in 100 mm culture dishes (Falcon; Becton Dickinson Labware, Franklin Lakes, NJ) in DMEM supplemented with 10% FCS. Twenty-four hours later, the cells were transfected with 5 µg of the hsa-miR-518c expression vector or the control vector (ORIGENE, Rockville, MD), using Lipofectamine LTX (Life Technologies, Carlsbad, CA) at the final concentration of 50 pM. The cells were incubated for 24 h in DMEM containing 10% FCS (V/V) and were subsequently trypsinized and seeded (1∶5 ratio) in 100 mm culture dishes in DMEM medium containing 10% FCS (V/V). Forty-eight hours later, the cells were placed in a selective medium containing Geneticin (700 µg/ml G418; Life Technologies). After selection with G418 for 2 weeks, all of the colonies were collected and the following stable transfectants were isolated: B88-518c, B88-mock2, CAL27-518c, and CAL27-mock cells.

### Quantitative RT-PCR

After 24 h, RNA was isolated from logarithmically growing cells with TRIzol reagent (Life Technologies) according to the manufacturer's instructions. Reverse transcription was performed by using the TaqMan MicroRNA RT Kit (Life Technologies) or miScript II RT Kit (Qiagen, Hilden, Germany). In quantitative PCR, miR-518c-5p and RNU6B miRNAs were detected using the TaqMan Gene Expression Assay (Life Technologies) or the miScript Primer Assay (Qiagen). Gene-specific products were measured continuously by an ABI StepOnePlus Real-Time PCR System during 40 cycles of PCR. In some experiments, cells were transfected with or without 50 nM miRCURY LNA microRNA inhibitor (Exiqon, Vedbaek, Denmark) using Lipofectamine RNAiMax (Life Technologies).

### MTT assay

Cells were seeded on a 96-well plate (Falcon; Becton Dickinson Labware) at 5×10^3^ cells per well in DMEM containing 10% FCS. Twenty-four hours later, the cells were transfected with or without 50 nM miRCURY LNA microRNA inhibitor using Lipofectamine RNAiMax. After 24 or 48 h, the number of cells was quantified by an assay using MTT [3-(4,5-dimethylthiazol-2-yl)-2,5-diphenyltetrazolium bromide; Sigma].

### *In vitro* cell migration assay

The *in vitro* migration of the cells was evaluated using transwells (Corning Corning, NY) as described previously [1]. The cells plugged in the pore or the cells attached to the lower surface of the membrane were counted in 10 fields at high power view (x 400) by a third person blinded to treatment conditions. In some experiments, 50 nM miRCURY LNA microRNA inhibitor was transfected before the cells were seeded on the upper chamber.

### Wound assay

At 24 h after seeding the cells, a linear wound was generated on the confluent monolayers by scraping with a pipette tip. Unattached cells were washed off with agitation. Cells were photographed at the same point on a grid 48 h later. Each cell line was plated and wounded in triplicate.

### Spheroid formation assay

Cells were seeded on a 96-well plate (NanoCulture plate; SCIVAX Life Sciences, Woburn, MA) at 1×10^3^ cells per well in DMEM containing 10% heat-inactivated FCS. Twenty-four hours later, the phenotype of the cells was observed by phase-contrast microscope (×300).

### Statistical analysis

Significant differences between the means for the different groups were evaluated with StatView 4.5 (Abacus Concepts, Berkeley, CA) using one-way ANOVA with significance set at p<0.05.

## Results

### Isolation of miR-518c-5p, which is induced by the SDF-1/CXCR4 system

**Figure 1:**
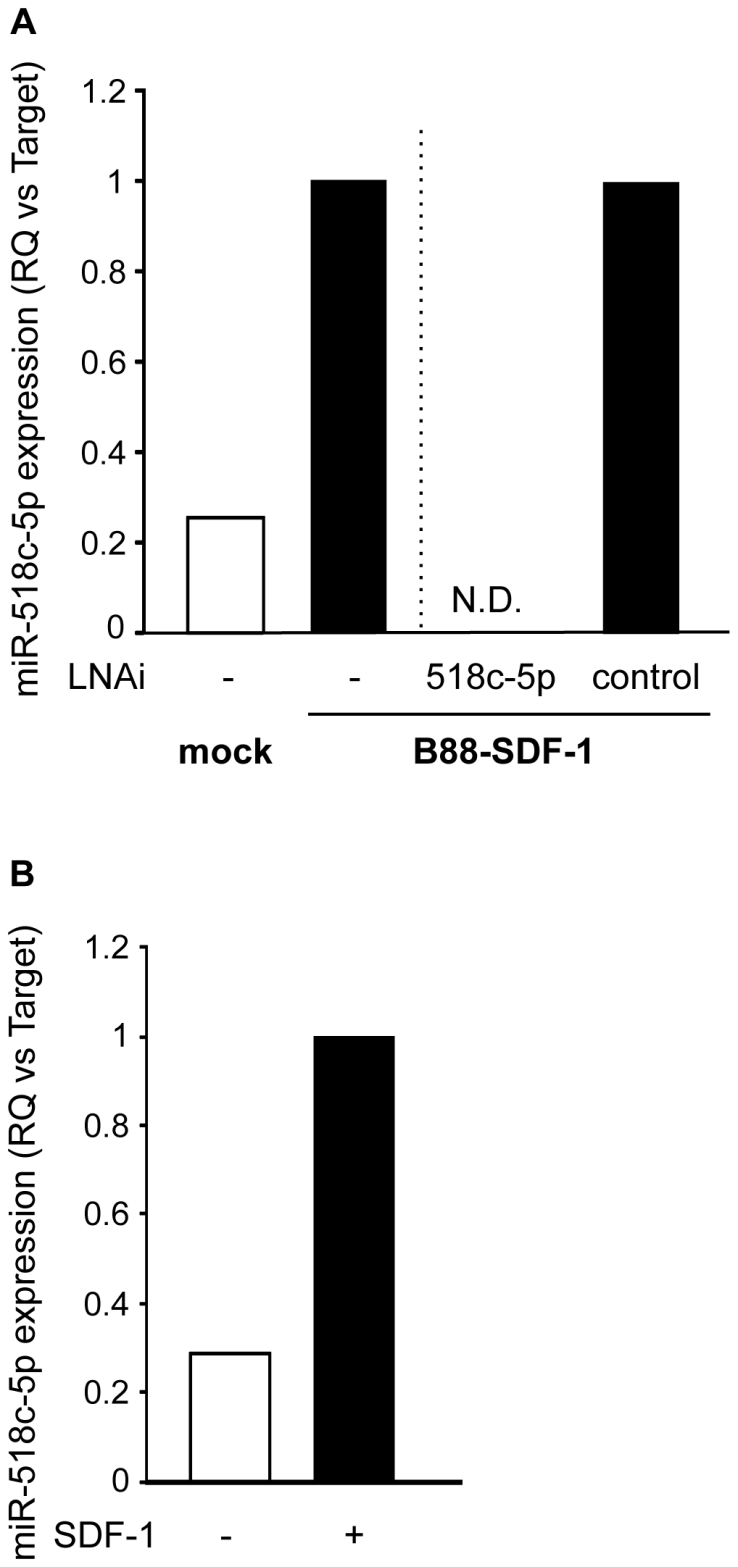
The upregulation of miR-518c-5p by the SDF-1/CXCR4 system in B88 cells. (A) The expression of miR-518c-5p was confirmed in B88-mock and B88-SDF-1 cells by real-time PCR. The downregulation of miR-518c-5p in B88-SDF-1 cells after treatment with a miR-518c-5p LNA inhibitor was also confirmed. N.D.: not detectable. (B) The expression of miR-518c-5p was confirmed in parental B88 cells after treatment with or without SDF-1 by real-time PCR.

We investigated miRNA downstream of the SDF-1/CXCR4 system using the oral cancer cell line, B88-SDF-1, which have an autocrine SDF-1/CXCR4 system and exhibit distant metastatic potential *in vivo* [4]. Microarray analysis revealed that several miRNAs were upregulated or downregulated in B88-SDF-1 cells compared to mock cells (data not shown). To confirm the specificity of the microarray analysis, the miRNA expression was confirmed by quantitative RT-PCR. Similar to the microarray results, miR-518c-5p (previously called miR-518c*) expression was upregulated in B88-SDF-1 cells compared to mock cells (Fig. 1A). Moreover, this upregulation was completely abrogated by the transfection of a miR-518c-5p LNA inhibitor (Fig. 1A). We also obtained similar results in the parental B88 cells stimulated with SDF-1 (Fig. 1B).

### Effect of miR-518c-5p inhibition on cell growth and SDF-1/CXCR4-dependent cell migration

**Figure 2:**
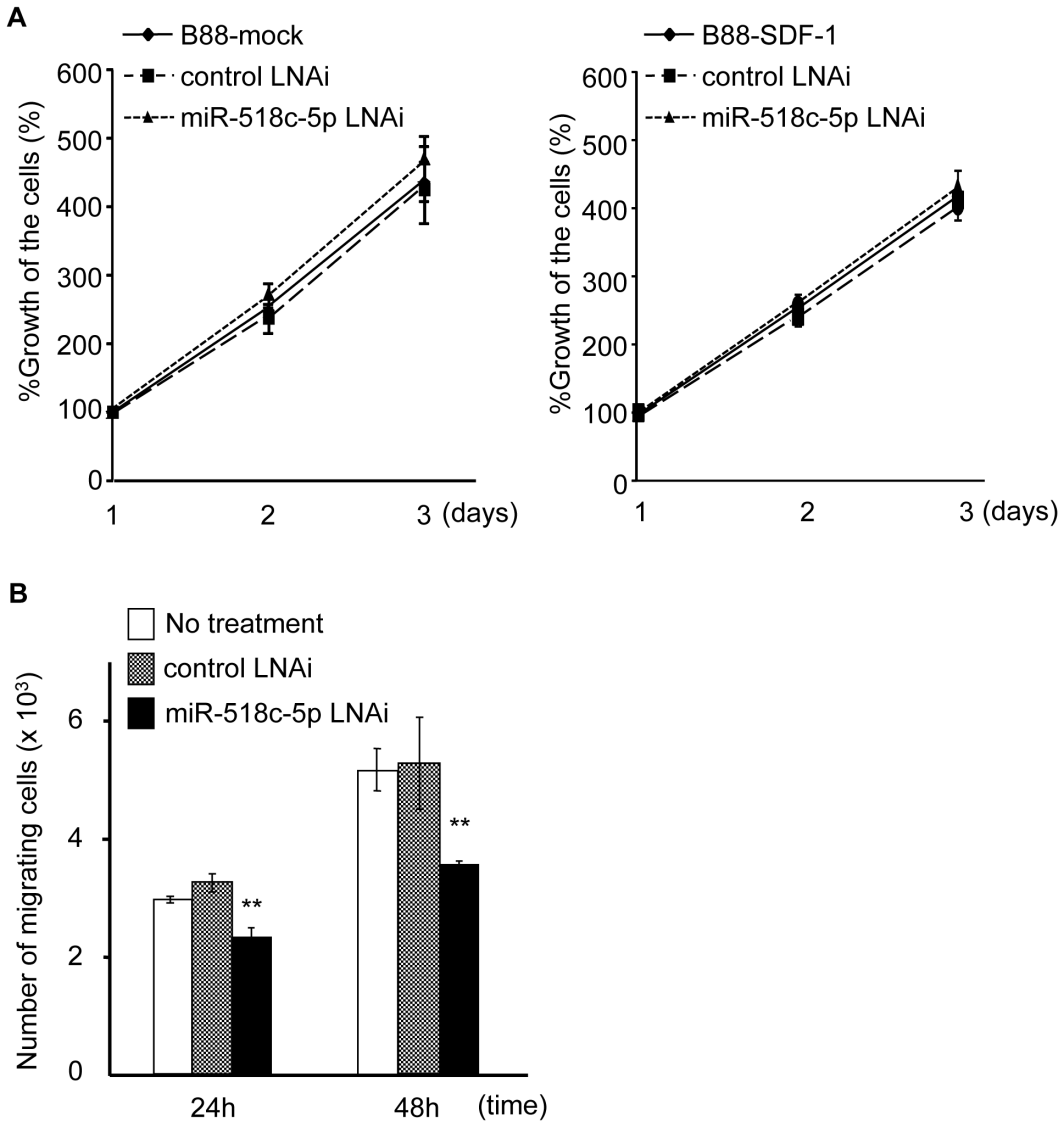
The involvement of miR-518c-5p on SDF-1/CXCR4-dependent cell migration. (A) The growth of B88-mock cells (left side) and B88-SDF-1 cells (right side) in the presence of either a control LNA inhibitor or a miR-518c-5p LNA inhibitor was examined using an MTT assay. There were no significant differences between the three groups by one-way ANOVA. (B) The motility of B88-SDF-1 cells in the presence of either a control LNA inhibitor or a miR-518c-5p LNA inhibitor was examined using a Transwell migration assay. A total of 2×10^4^ cells were transfected before being seeded in the chamber. **; *p*<0.01 when compared to untreated control or control LNA inhibitor-treated cells by one-way ANOVA.

Previously, we found that neither the paracrine nor the autocrine SDF-1/CXCR4 system affected the anchorage-dependent growth of B88 cells [1], [4]. We examined the effect of miR-518c-5p on cell growth by using a specific miR-518c-5p LNA inhibitor. The inhibitor did not affect the growth of either B88-mock or B88-SDF-1 cells (Fig. 2A). We next investigated the effect of miR-518c-5p on the SDF-1/CXCR4-dependent migration of the cells. Migration chamber assays revealed that the enhanced motility of B88-SDF-1 cells was significantly impaired after treatment with a miR-518c-5p LNA inhibitor (Fig. 2B).

### Effect of miR-518c-5p overexpression on cell growth

**Figure 3:**
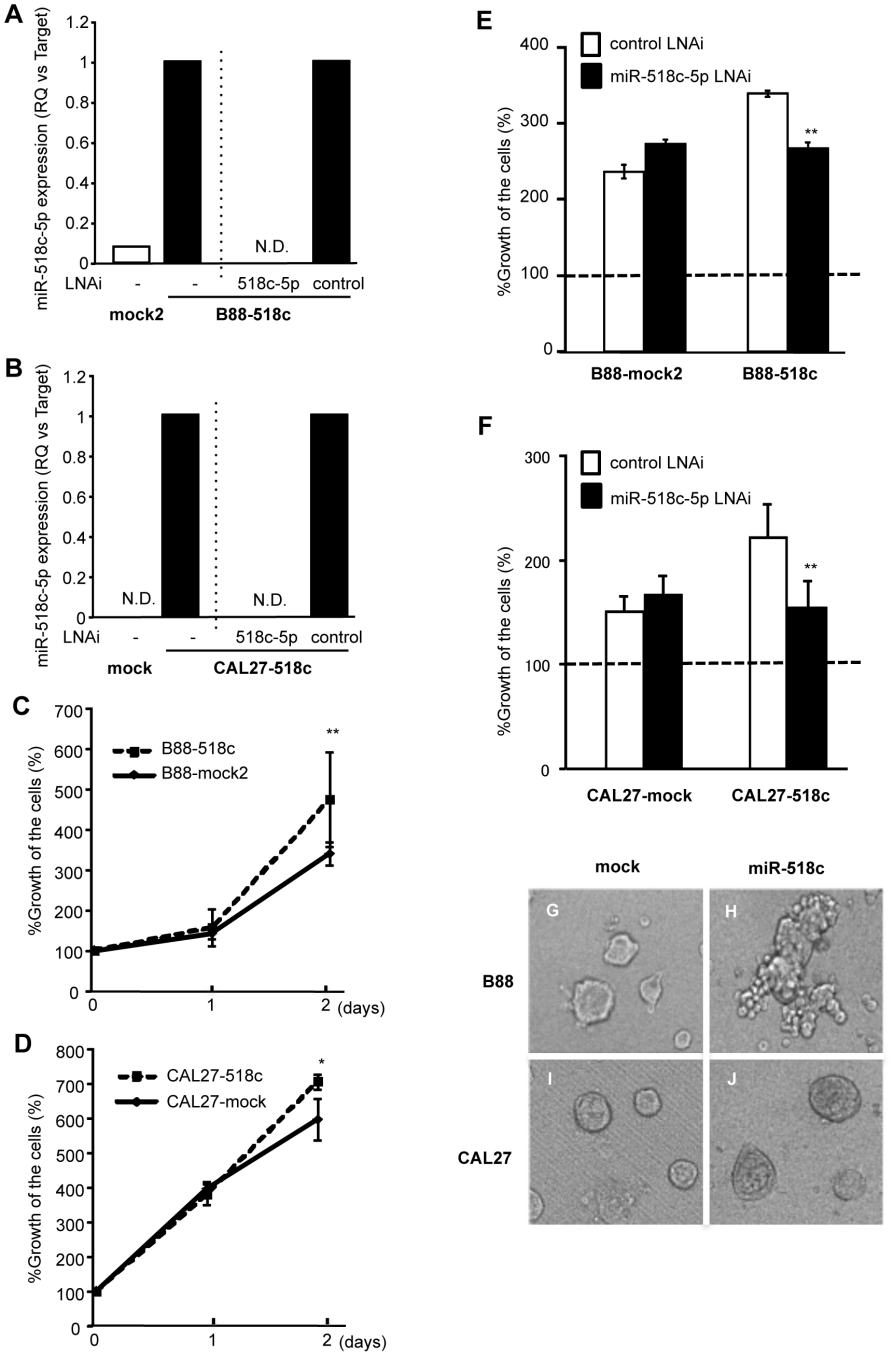
miR-518c-5p overexpression induced the growth of oral cancer cells. B88 cells and CAL27 cells were stably transfected with a control vector or a miR-518c expression vector, and B88-mock2 or B88-518c cells and CAL27-mock or CAL27-518c cells were isolated, respectively. (A, B) The expression of miR-518c-5p in the B88-transfectants (A) and CAL27-transfectants (B) were assessed by real-time PCR. The downregulation of miR-518c-5p in B88-518c cells or CAL27-518c cells after treatment with a miR-518c-5p LNA inhibitor was also confirmed. (C, D) Anchorage-dependent growth of the B88-transfectants (C) and CAL27-transfectants (D) were evaluated using an MTT assay. *; *p*<0.05, **; *p*<0.01 when compared to mock cells by one-way ANOVA. (E, F) The growth of the B88-transfectants (E) and CAL27-transfectants (F) in the presence of either a control LNA inhibitor or a miR-518c-5p LNA inhibitor was examined using an MTT assay. *; *p*<0.05, **; *p*<0.01 when compared to untreated control or control LNA inhibitor-treated cells by one-way ANOVA. (G-J) Anchorage-independent growth of B88-mock2 (G), B88-518c (H), CAL27-mock (I) or CAL27-518c (J) was evaluated using a nano-culture plate.

Next, we performed a gain of function assay overexpressing miR-518c-5p in parental B88 cells and also in parental CAL27 cells in which the expression of CXCR4 and miR-518c-5p was not detectable. After transfection of the empty vector or miR-518c expression vector, we collected all of the G418 resistant clones to avoid clonal heterogeneity of the cells and established the following transfectants, B88-mock2, B88-518c, CAL27-mock and CAL27-518c. We could detect the upregulation of miR-518c-5p in the miR-518c transfectants compared with mock cells, which was completely inhibited by treatment with the miR-518c-5p LNA inhibitor (Fig. 3A,B). The anchorage-dependent growth potential of both the B88-518c and CAL27-518c cells was significantly enhanced compared with that of the mock cells (Fig. 3C,D), which were significantly inhibited by treatment with the miR-518c-5p LNA inhibitor (Fig. 3E,F). Next, we examined the effect of miR-518c overexpression on the anchorage-independent growth of the cells. B88-mock2 cells formed round and tight cell-cell adhesion spheroids (Fig. 3G), whereas B88-518c cells formed grapevine-like spheroids (Fig. 3H). In contrast, both CAL27 transfectants formed round spheroids (Fig. 3I,J), but larger spheroids were observed in CAL27-518c cells (Fig. 3J).

### Effect of miR-518c-5p overexpression on cell migration

**Figure 4:**
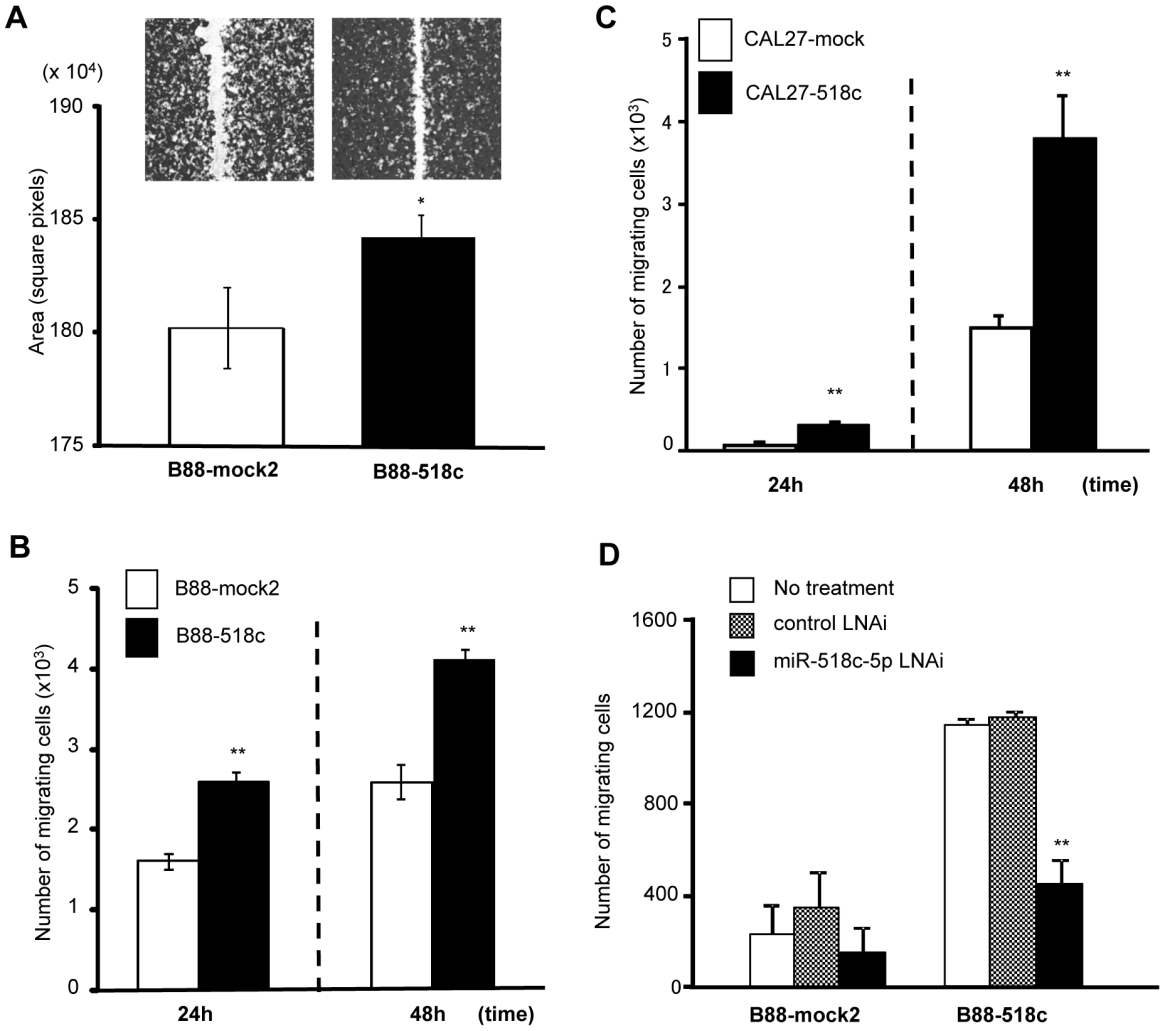
miR-518c-5p overexpression also induced the migration of oral cancer cells. (A) B88-mock2 or B88-518c cells were cultured to confluence. A wound healing assay was performed. *; *p*<0.05 when compared to mock cells by one-way ANOVA. (B,C) The motility of B88-518c cells (B) or CAL27-518c cells (C) was evaluated using a Transwell migration assay. Each transfectant was seeded at a density of 1×10^4^. **; *p*<0.01 when compared to mock cells by one-way ANOVA. (D) The motility of B88-transfectants in the presence of either a control LNA inhibitor or a miR-518c-5p LNA inhibitor was also examined using a Transwell migration assay. **; *p*<0.01 when compared to untreated control or control LNA inhibitor-treated cells by one-way ANOVA.

B88-518c cells acquired a significant chemokinetic response (Fig. 4A) and enhanced cell motility (Fig. 4B). We also detected an enhanced migration in the CAL27-518c cells (Fig. 4C). The enhanced cell motility of the B88-518c cells was significantly impaired after treatment with the miR-518c-5p LNA inhibitor (Fig. 4D).

### The role of miR-518c-5p on the growth and metastasis *in vivo*

**Figure 5:**
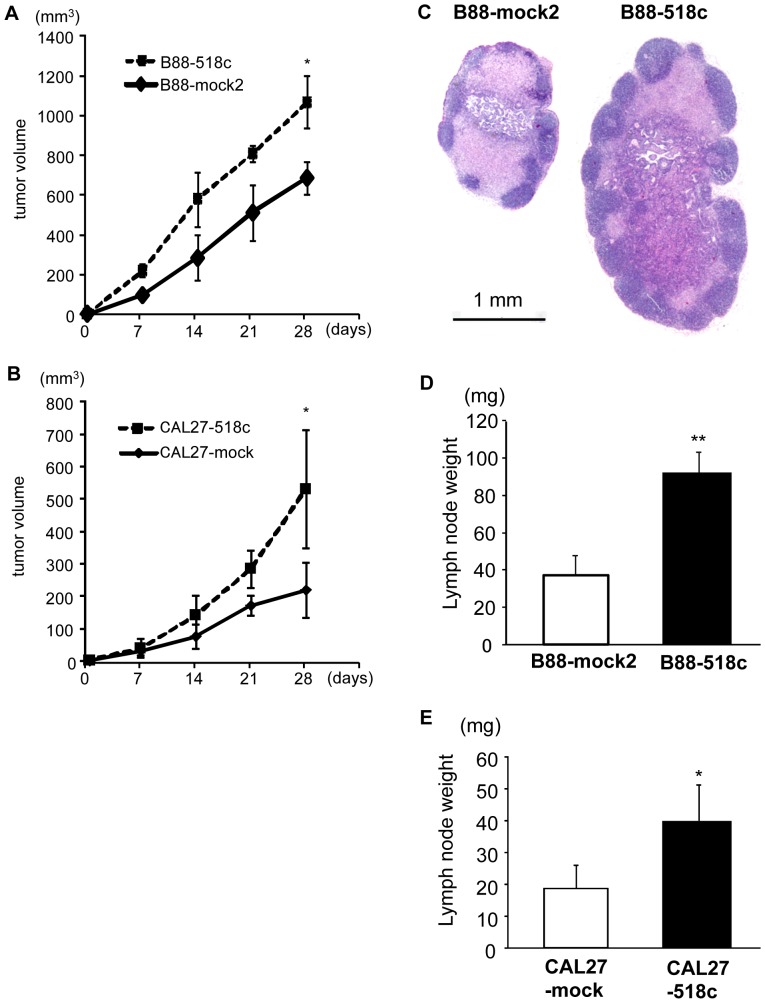
The enhancement of tumor growth and lymph node metastasis in miR-518c transfectants. (A, B) B88-transfectants (A) and CAL27-transfectants (B) were orthotopically inoculated into the masseter muscle of nude mice, which were sacrificed at day 35. The size of primary tumors was measured once a week. The results presented are the means ± SD. *; p<0.05 when compared to mock cells by one-way ANOVA. (C) Representative H&E staining of the metastatic lymph nodes from the nude mice inoculated with B88-mock2 cells (left) and B88-518c cells (right). (D, E) The weight of the submandibular lymph nodes was measured in the mice inoculated with B88-transfectants (D) and CAL27-transfectants (E). *; p<0.05 when compared to mock cells by one-way ANOVA.

**Figure 6:**
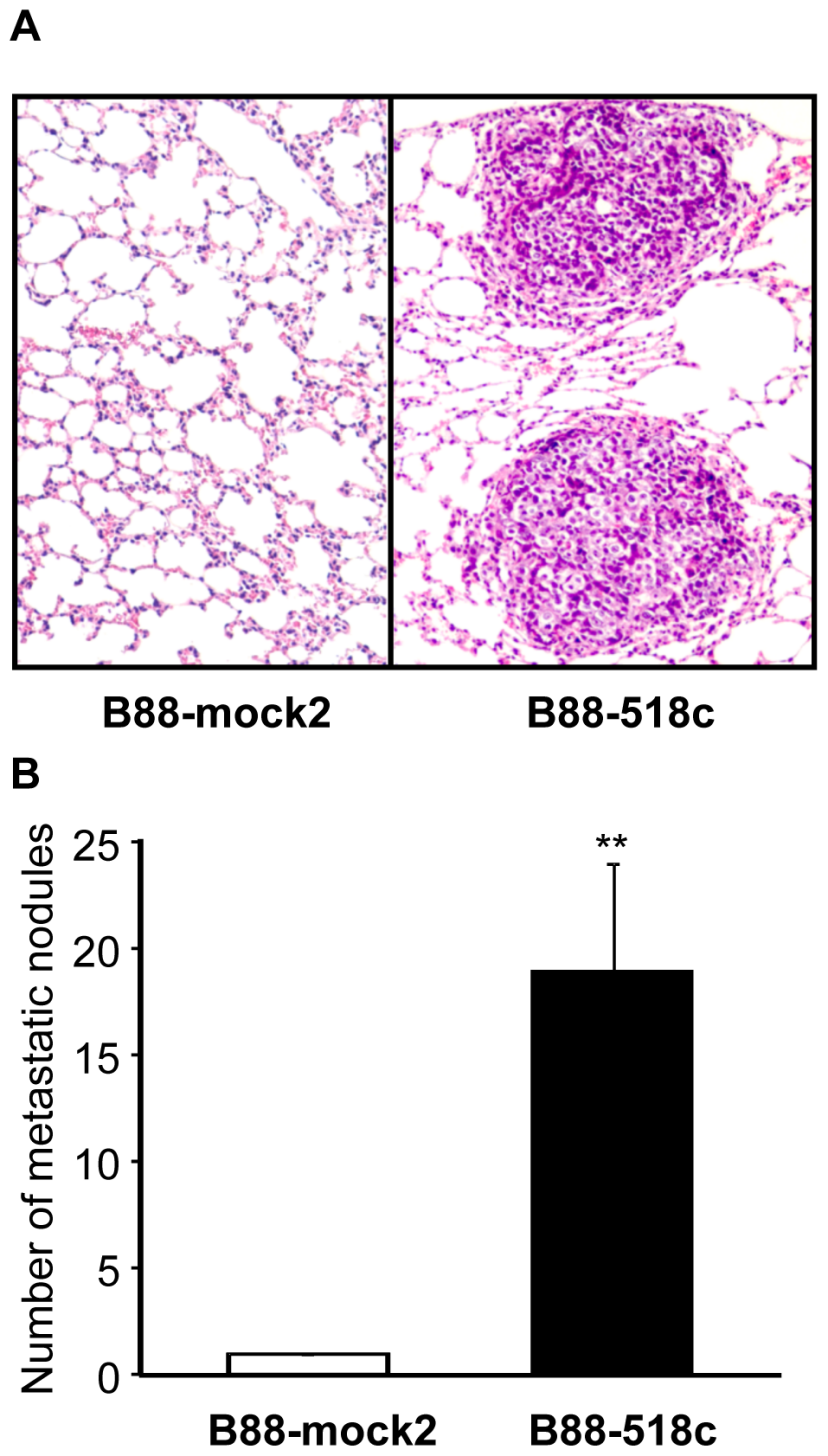
The enhancement of lung metastases in miR-518c transfectants. Cells were inoculated into the blood vessels of nude mice, which were sacrificed at day 35. (A) Representative H&E staining of the lungs from the nude mice inoculated with B88-mock2 cells (left) and B88-518c cells (right). (B) The metastatic nodules were counted under a microscope. **, p<0.01 when compared to mock cells by one-way ANOVA.

We next evaluated the effect of miR-518c-5p on growth and metastasis in vivo. B88 and CAL27 transfectants were orthotopically inoculated into the masseter muscle of nude mice [1]. The size of the primary tumor from B88-518c cells (Fig. 5A) and CAL27-518c cells (Fig. 5B) was significantly increased compared to tumors from mock cells. Although both mock and miR-518c transfectants histopathologically metastasized to the cervical lymph nodes, the weight of lymph nodes containing miR-518c transfectants was significantly heavier than that of lymph nodes containing mock cells (Fig. 5C–E). We next performed intravenous inoculation using these transfectants. Numerous and large metastatic nodules were detected in the lungs in all of the mice inoculated with B88-518c cells (Fig. 6A). The number of nodules from mice inoculated with B88-518c cells was significantly increased compared with the number from mock cells (Fig. 6B).

## Discussion

miRNAs are small, endogenous, evolutionarily conserved non-coding RNAs that regulate approximately 60% of mammalian genes by modulating their transcript levels. miRNAs are involved in many molecular pathways and in pivotal biological processes including cell growth, development, differentiation, proliferation and cell death [11]. Recent evidence has demonstrated the role of miRNAs in modulating the metastatic process in the context of solid tumors, and these miRNAs have been termed metastamir [13]. Numerous metastamirs have been identified and have pro- and anti-metastatic effects [11]. However, it is likely that there are many miRNAs that are metastamir with unknown functions, because 3% of the human genome is estimated to code for miRNA sequences [14]. In the present study, we demonstrated that miR-518c-5p may be a novel metastasis-promoting metastamir. miR-518c-5p was originally identified in the 250 small RNA libraries from 26 different organ systems and cell types of human and rodents, enriched in neuronal as well as normal and malignant hematopoietic cells and tissues [15]. Although the function of miR-518c-5p has not been clearly defined in cancers, Dyrskjøt and colleagues demonstrated that miR-518c-5p was significantly associated with disease progression when correcting for disease stage and grade using a multivariate Cox regression analysis [16]. Furthermore, recent investigations showed that miR-518c-5p exhibited a significant upregulation in retinoblastoma in a miRNA microarray analysis [17]. Taken together with our present results, miR-518c-5p may function as an oncomiR in tumor cells.

In the present study, we showed that miR-518c-5p is upregulated by the SDF-1/CXCR4 system in a paracrine or autocine manner. Rhodes and colleagues investigated miRNA expression induced by the SDF-1/CXCR4 system in estrogen receptor-alpha-positive breast cancer cells [18]. However, neither miR-518c-5p nor another miRNA detected in our miRNA analysis were present in their results. Furthermore, although we also analyzed the miRNA microarray analysis in the CXCR4-positive salivary gland cancer cells, ACC-M [19], after stimulation with SDF-1, there were not any common miRNAs between the three miRNA microarray analyses despite the use of the SDF-1/CXCR4 system in these cancer cells (data not shown). This finding may be due to the different experimental conditions of the cells; however, it may be due to the different site-specific metastatic pattern of these cancer cells. For example, breast cancer favors metastasizing to the bone, salivary gland cancer to the lung, and oral cancer to the lymph node. Thus, miRNAs that are necessary for homing to the specific target organ might be activated by CXCR4 upon binding of its ligand, SDF-1 in these cancer cells.

The mechanism of miR-518c-5p upregulation by the SDF-1/CXCR4 system in oral cancer cells was not clarified in the present study. miR-518c-5p belongs to the miR-515 family in a chromosome 19 miRNA cluster, so called C19MC [20]. C19MC is the largest cluster, conserved only in primates, encoding 59 mature miRNAs and displaying a very low expression in most human tissues due to epigenetic control [20]. Because the SDF-1/CXCR4 system can upregulate DNMT1/DNMT3ß expression [21] or ANP32A/Lanp, a component of the inhibitor of histone acetyl transferase [22], miR-518c-5p upregulation may be dependent on the result of demethylation of C19MC. Alternatively, recent investigations demonstrated that some of the miRNA members in C19MC were upregulated in hepatocellular carcinoma characterized by aberrant IGF, phosphatidylinositol 3 kinase (PI3K)-Akt-mTOR or p53 pathway activation [23], [24]. The SDF-1/CXCR4 system could also activate the PI3K-Akt pathways in B88 cells [1], and these pathways might be involved in the upregulation of miR-518c-5p. We further examined miR-518c-5p expression using an oral cancer cell line, HNt, in which the expression of CXCR4 was 7.5-fold lower than that in B88 cells [1], [3]. HNt-SDF-1 cells did exhibit slight, but not significant, phenotypic changes *in vitro* and *in vivo*, in which the activation of PI3K-Akt by SDF-1 was not detectable [4]. Furthermore, the miR-518c-5p induction in HNt-SDF-1 cells was detectable only at a marginal level, most likely due to the reduced expression of CXCR4, compared with that of B88 cells (data not shown). Thus, the CXCR4 expression level and strong downstream signaling, such as PI3K-Akt, might also be critical for the activation of miR-518c-5p expression, therefore, further studies would be needed to clarify this mechanism.

In the present study, miR-518c-5p induced by the SDF-1/CXCR4 system did not stimulate cell growth but did enhance cell migration using an LNA inhibitor. In contrast, we could detect both growth stimulation and enhanced-migration after the transfection of a miR-518c expression vector, *in vitro* and *in vivo*. Although the reason is unclear, the finding may be due to the effect of miR-518c-3p produced by the miR-518c expression vector. However, we could not find previous reports about miR-518c-3p in cancer cells and cell growth, and an LNA inhibitor against miR-518c-5p could suppress the enhanced growth both in B88-518c and CAL27-518c cells (Fig. 3E,F). Thus, we believe that the effect of miR-518c-3p on the growth of the cells could be partially neglected. The second possibility may be due to the downregulation of the nonphysiological or false target mRNAs that share a similar seed sequence by the overexpression of miR-518c-5p. However, overexpression of miR-518c-5p by the oligonucleotide contrarily inhibited the growth and migration of the B88 cells and CAL27 cells, most likely due to the downregulation of false target mRNA (data not shown). This result indicates that expression of miR-518c using the vector method induced a physiological and non-toxic condition in these oral cancer cells. Thus, we believe that the moderate induction of miR-518c-5p may be sufficient for cell migration, but strong induction of miR-518c-5p may be needed for the induction of cell growth. Because the *in silico* mRNA targets of miR-518c-5p include several types of genes that regulate growth and metastasis (S1 Table), theses target genes may control the different effect of miR-518c-5p.

C19MC, including miR-518c-5p, maps to chromosomal band 19q13.4 [20]. Kasamatsu and colleagues demonstrated that the 19q13.4 locus is amplified in salivary gland adenoid cystic carcinoma, a highly metastatic type of head and neck cancer [25]. Moreover, the amplification of this locus is frequently detected in ependymoblastoma and embryonal tumors with abundant neuropil and true rosettes, very aggressive embryonal neoplasms, at a rate of 93% [26]. The SDF-1/CXCR4 system plays major roles in maintaining the architecture of niches for hematopoietic and other tissue stem cells such as germ cells and follicular, intestinal, and neural stem cells [27], [28]. Notably, most cancer stem cells (CSCs) express CXCR4 and respond to a chemotactic gradient of SDF-1, suggesting that CSCs most likely represent a subpopulation capable of initiating metastasis [29]. In addition, several groups have recently linked the expression of members of C19MC with the miRNA signature characteristic for human embryonic stem cells [20]. Taken together, C19MC, including miR-518c-5p, might be expressed in CSCs that are involved in growth and metastasis.

## Conclusions and Recommendations

These results indicated that miR-518c-5p regulates the growth and metastasis of oral cancer as a downstream target of the SDF-1/CXCR4 system. In the future, it would be critical to examine the association between CXCR4 and miR-518c-5p expression in the clinical material. If the molecular mechanism of miR-518c-5p against cancer metastasis could be further clarified, the suppression of cell growth and migration by a miR-518c-5p inhibitor may serve as a basis for the development of therapies against metastasis.

## Supporting Information

S1 Table **miRNA targeted by miR-518c-5p is analyzed by use of microRNA.org program (** http://www.microrna.org/microrna/getMirnaForm.do **), and the cut-off value is adapted less than -1.5 of miRSVR score.**
(DOC)
