# Supplementary material for: Involvement of miR-518c-5p to Growth and Metastasis in Oral Cancer
Source: PLoS One. 2014 Dec 23;9(12):e115936. doi: 10.1371/journal.pone.0115936 (PMC4275267; doi:10.1371/journal.pone.0115936)
Supplement: S1 Table — miRNA targeted by miR-518c-5p is analyzed by use of microRNA.org program ( http://www.microrna.org/microrna/getMirnaForm.do ), and the cut-off value is adapted less than -1.5 of miRSVR score. (DOC) [file pone.0115936.s001.doc]

Table S1. In silico analysis of miR-518c-5p target mRNA by microRNA.org

| **mRNA targeted by miR-518c-5p** | **mirSVR score** | **Refseq ID** |
| --- | --- | --- |
| **SFTPA1** | -2.84 | BC026229 |
| **ARHGAP25** | -2.82 | BC039591 |
| **TFAP2D** | -2.41 | BC144200 |
| **MICALCL** | -2.38 | NM_032867 |
| **SGMS1** | -2.32 | NM_147156 |
| **USP15** | -2.19 | NM_006313 |
| **CD40LG** | -2.16 | NM_000074 |
| **CDK1** | -2.13 | AK295741 (alternative isoform) |
| **ASB9** | -2.09 | NM_024087 |
| **CCDC30** | -2.04 | AK302075 (alternative isoform) |
| **DYNLRB2** | -2.03 | BC054892 (alternative isoform) |
| **ST8SIA5** | -2.02 | AK310764 (alternative isoform) |
| **HS3ST5** | -2.02 | NM_153612 |
| **PIP5K1B** | -1.86 | BC030587 |
| **WDR61** | -1.85 | AK304617 (alternative isoform) |
| **FLJ41200** | -1.8 | AK123194 |
| **NCRNA00093** | -1.8 | NR_024130 |
| **AKR1E2** | -1.78 | AB055603 (alternative isoform) |
| **PIWIL3** | -1.73 | NM_001008496 |
| **CDK5RAP1** | -1.72 | AK095644 (alternative isoform) |
| **SERPINB5** | -1.71 | NM_002639 |
| **GIT2** | -1.69 | AK304240 (alternative isoform) |
| **TAF12** | -1.68 | NM_005644 |
| **HYALP1** | -1.64 | NR_002731 |
| **MEST** | -1.64 | NM_002402 |
| **HOXC10** | -1.62 | NM_017409 |
| **FLJ22536** | -1.62 | AK022865 |
| **ELP4** | -1.62 | CR610736 (alternative isoform) |
| **CARD6** | -1.61 | NM_032587 |
| **CD34** | -1.59 | NM_001025109 |
| **FBXO21** | -1.58 | NM_015002 |
| **TAF1B** | -1.58 | NM_005680 |
| **SH3KBP1** | -1.57 | NM_031892 |
| **ELP2** | -1.55 | AF403223 |
| **DUXAP10** | -1.55 | AK022914 |
| **LOC100131060** | -1.55 | AK296298 |
| **LRRC7** | -1.54 | AF498274 |
| **LOC203274** | -1.52 | BC080605 |
| **DDX52** | -1.52 | NM_007010 |
| **SLC44A1** | -1.51 | AJ272365 |
| **NLRP5** | -1.5 | NM_153447 |
| **KIAA1804** | -1.5 | NM_032435 |
